# Remote Consultations in England During COVID-19: Challenges in Data Quality, Linkage, and Research Validity

**DOI:** 10.2196/66672

**Published:** 2025-08-20

**Authors:** Liliana Hidalgo-Padilla, Massar Dabbous, Kristoffer Halvorsrud, Thomas Beaney, Gideon Gideon, Eoin Gogarty, Geva Greenfield, Benedict Hayhoe, Gabriele Kerr, Rosalind Raine, Nirandeep Rehill, Robert Stewart, Fiona Gaughran, Mariana Pinto da Costa

**Affiliations:** 1 Institute of Psychiatry, Psychology and Neuroscience, King's College London, 16 De Crespigny Park, London, SE5 8AF, United Kingdom, 44 020 7848 0002; 2 Department of Primary Care and Population Health, University College London, London, United Kingdom; 3 Department of Primary Care and Public Health, Imperial College London, London, United Kingdom; 4 South London and Maudsley NHS Foundation Trust, London, United Kingdom

**Keywords:** telemedicine, telecare, remote consultations, data sources, telehealth, remote consultation, electronic health record, EHR, remote monitoring, England, data quality, secondary care, COVID-19

## Abstract

The COVID-19 pandemic accelerated the adoption of remote consultations across health care, requiring rapid adjustments in service delivery. Consequently, there is an urgent need to understand the impact of remote consultations on health pathways. This viewpoint paper explores key challenges in data sources in England that hinder research on the impact of remote consultations on health outcomes. Based on our experience conducting research on this topic, we present variations in observational study findings and their validity, considering differences in population characteristics and data sources. We provide recommendations to enhance data quality for future research, including improvements in data recording platforms and strengthened structures for linking primary and secondary care electronic health records.

## Introduction

The need to protect patients and health care staff during the COVID-19 pandemic accelerated the use of remote consultations in most health service settings [1-4]. This shift allowed medical appointments to be conducted remotely rather than in person, offering greater flexibility and convenience to both patients and providers [5]. Observational studies have examined the rate and impact of the shift to remote consultations during the COVID-19 pandemic across different populations and services, including mental health [6-9], oncology [10-14], dermatology [15,16], respiratory [17], orthopedics [18], older adult care [19], primary care [20], general outpatients [21], and COVID-19 inpatient care [22]. In the National Health Service (NHS) in England, a significant proportion of patient interactions continues to be conducted remotely despite the gradual reintroduction of face-to-face consultations.

Remote consultations have the potential to improve health care access, particularly for patients living in rural areas or those with limited mobility and constrained time availability, and to enhance efficiency amid increasing resource constraints. Although remote consultations are generally acceptable and convenient for both patients and health care professionals, there are disadvantages, such as the loss of nonverbal communication, inability to perform physical examinations, increased workloads for health care professionals, and insufficient institutional resources to support remote care effectively [3,23-26]. Understanding the long-term impact of remote consultations is crucial for developing strategies that optimize their benefits while addressing these limitations.

While evidence exists about the extent of ongoing health service provision for remote consultations and its impact on patient and health service outcomes, research in this area is hindered by inconsistencies in data recording, availability, quality, and reliability, as well as by variations in populations and methodologies, and difficulties linking primary and secondary care records.

The “Digital transformation in the NHS” strategy [27] was introduced to address these challenges by building knowledge from previous initiatives of technology implementation within the NHS in England. This strategy emphasizes both the active engagement of staff and the critical need for up-to-date and reliable data to measure the impact of remote consultations on patient and health care outcomes. This will enable planning for improvements, address gaps, and expand or decommission service components, as appropriate, both across the NHS and in other health care settings.

In this viewpoint paper, we highlight challenges in the quality and linkage of electronic health record (EHR) infrastructures in NHS England, including inconsistencies in data documentation, interoperability issues, and limitations in data linkage between primary and secondary care. Additionally, we discuss variations in findings due to differences in population characteristics, service settings, and outcome measures. We also explore the uptake and engagement with remote consultations and the lack of long-term evaluations and consider the implications for future service planning. This paper stems from discussions among research teams from 3 leading universities in London, United Kingdom, who collaborated to explore the application of technology in the health care sector [28]. This viewpoint draws on our expertise working on data linkage across 2 geographical areas to evaluate the impact of remote consultations in different UK settings and on our systematic review of the relevant literature. While our focus is on England, similar issues may arise in other health care systems worldwide.

## Data Challenges in Remote Consultations

### Inconsistent and Incomplete Data Documentation

Complete, accurate, and consistent documentation is fundamental to ensure the quality and reliability of EHR-derived observational data. However, data recording inconsistencies pose a recurring challenge. Missing or variably recorded information can result from different factors such as time pressures during consultations, differences in clinician recording habits, and variations in health care settings [29]. For example, a study found that the frequency of diagnostic coding for long-term conditions was associated with patient sociodemographic characteristics, general practitioner (GP) practice—probably due to organizational processes—and disease inclusion in the Quality and Outcomes Framework financial incentive program [30]. However, it is hard to determine whether these associations are due to clinicians’ biases or unmeasured confounding variables.

Additionally, large amounts of patient data are recorded as “free text” rather than structured data, making them difficult to analyze for research, service evaluation, and clinical audit. While natural language processing (NLP) techniques allow to extract valuable insights from free-text data [29,31], their effectiveness is limited by cross-system inconsistencies across EHR infrastructures and NHS trusts [32]. For example, NLP models need to be revalidated when used across other platforms due to different local documentation and recording practices. While the use of standardized and structured data (eg, predefined categories and drop-down boxes) is more amenable to analysis, it is insufficient because this does not guarantee improved accuracy or consistency between raters. Different platforms may offer different categorizations and therefore result in worse between-site consistency than NLP-processed clinical text [17].

Many of these challenges have been recognized as barriers in health care data research. However, the rapid shift to remote consultations during COVID-19 exacerbated these issues, with long-lasting effects [33]. Clinicians, navigating considerable operational pressures in the pandemic context, could have had less time to record properly structured data, leading to relying on free-text notes instead of structured coding.

### EHR Interoperability Issues

The use of a plethora of different software and digital platforms for remote consultations with varying degrees of interoperability with EHRs also represents a challenge to deriving comparable data. Various software solutions used across NHS services often lack integration, preventing data exchange between primary, secondary, and community care settings. For example, discrepancies across different EHR providers could have led to inconsistent documentation of conditions such as long COVID, many of which were clinical- or service-related, making it difficult to derive meaningful comparisons. A study using population-based data in primary care from the 2 largest platforms in England (EMIS and TPP) [34] showed a higher number of people diagnosed with long COVID in London compared to the East of England. This regional variation could be associated with the differences in the user interfaces of both EHR providers. In addition, there was an increase in long COVID diagnoses in January 2021, probably due to the increase in awareness of this condition among clinicians and the inclusion of the diagnostic codes in at least 1 of the platforms.

Notably, the urgency of the COVID-19 pandemic led to the rapid adoption of new digital tools and platforms to facilitate remote consultations. While these systems addressed immediate needs, they often lacked full integration with existing EHR infrastructures, creating long-term challenges for data quality and interoperability.

### Lack of Standardized Definitions for Remote Consultations Across Systems

Another significant barrier to evaluating remote consultations is the lack of standardization across platforms. Differing variable definitions can result in incomplete or incomparable data and pose a barrier to deriving meaningful findings. Definitions for what constitutes a remote consultation usually include telephone or videoconferencing interactions [6-8,18], but in some cases, it also considers email [17] or e-consultations [12], leading to discrepancies in data classification. Additionally, information is not always consistently recorded across systems, which may not allow a thorough understanding of events. For example, the inability to distinguish between different modes of psychiatric remote consultations (video, telephone, email, and SMS text message) hindered the possibility of exploring how clinicians performed clinical tasks based on the means of communication used [7], which might affect the quantity of information about a patient’s “real-time” environment.

### Difficulties in Data Linkage

Effective data linkage is essential to track patient journeys across primary and secondary care and assess the impact of remote consultations on their outcomes. However, in England, health care sectors use a wide range of EHR platforms, hence making linkage challenging [35,36]. The establishment of a network of Secure Data Environments aims to improve access to NHS data through shared and secure working practices, including sharing methods and coding [37]. This has been shown to be beneficial in studies linking primary and secondary care data to identify severe mental illness characteristics associated with cardiovascular diseases [38] but has been insufficient to compare attendance rates between remote and in-person consultations because modality was not available in primary care data [28].

Linkage of routine data resources can be complex, time-consuming, and expensive to establish. In part, this is because of the need for robust information governance. Without cross-system linkages of comparable data, it is difficult to assess long-term outcomes. For example, one study showed an increase in attendance rates to remote mental health services in South London, but the number of prescriptions of antipsychotics and mood stabilizers did not change [7]. However, one of the study limitations was that it did not use linkages to primary care data, which would be essential to have a more comprehensive understanding of the variations in prescription levels.

Data coverage and representativeness are limited when not all data can be linked. For example, a study examining primary care consultations for respiratory tract infections, using a large dataset covering 160 GP practices, only included patients with complete consultation notes and linked hospital records; thus, the authors warn that characteristics and outcomes of excluded patients may differ [17].

## Consequences of Data Limitations on Research Quality

### Variations in Study Findings

Similar research questions can sometimes yield conflicting results due to differences in data quality. This variability can be explained by differences in recording practices at the organizational (eg, funding and trust priorities), team (eg, staff levels and expertise), and individual patient and clinician levels (eg, number of sites, sample size, sociodemographic and clinical characteristics, and social disadvantage of the population). While the results are valid in each context, variability in the remits of nominally similar services all impact the interpretation and, thus, the generalizability of findings.

This can be illustrated by variations in the uptake of remote consultations among older people. Studies in mental health services in South London [7] and in primary care across England [19] during the pandemic demonstrated lower uptake of remote consultations among people aged 65 years and older compared to younger age groups. While this might suggest a general preference for face-to-face over remote among older patients or lack of access to technology, another study of patients with dementia in 2 mental health trusts reported fewer missed appointments in those attending remotely compared to face-to-face [8], which might be due to a more frequent presence of caregivers helping people with dementia in the use of technology.

Uptake could have also changed due to other patient characteristics across GP practices in England. Trajectories of 21 GP practices in Bristol, North Somerset, and South Gloucestershire between April-July 2019 and April-July 2020 varied by patient age (eg, no change for people aged 70 years and older), mental health status (increased among patients with poor baseline mental health and decreased among patients with good baseline mental health), and shielding status (increased in shielding patients and decreased in nonshielding patients) [20].

In terms of diagnostic accuracy, one study in primary care reported that remote consultations were sufficient to address most patient problems [20], but another study concluded that simplified digital consultation tool interactions with primary care patients could be only limited to low-risk queries [39]. The contrast in these findings might be secondary to differences in the study population (eg, patients from different regions of England) and in the data (eg, different data points and number of GP practices included). Furthermore, a study in secondary care settings analyzing oncology consultations reported that remote assessments were useful for routine follow-ups but insufficient for initial cancer diagnoses due to the lack of physical examinations [13].

Differences in outcomes may be attributable to different clinical and social needs when receiving care. For example, 3 studies explored remote triaging in 41 secondary care head and neck cancer services [14], 2 maternity services in hospital trusts [40], and 154 GP practices [41]. The triage in the head and neck cancer and maternity services, using data from May to August 2020 and October 2021 to February 2022, respectively, found that the remote triage was effective to identify the level of severity of risk cases, which was beneficial for the patients’ care management, provided reassurance for staff, and alleviated infrastructural pressures. However, the study in primary care using prepandemic data did not find any differences in the assessment outcomes nor the time or burden to the practitioners. These differences might be explained by the impact of the context of the pandemic on both staff and patients, but also on the diverse needs for each type of service. Without comprehensive and standardized data, it is difficult to assess whether remote consultations lead to comparable patient outcomes as in-person visits.

Some studies suggest that remote consultations during the COVID-19 pandemic increased access to health care by reducing travel burdens [42-44] and improving attendance rates [45,46]. It was further suggested that insofar as the purpose was to exchange explicit and less emotionally loaded health care information, the use of telephone was particularly frequent, including check-up calls to patients [44,47]. Although these studies provide helpful discourses of facilitators and challenges during the implementation of remote consultations during the COVID-19 pandemic, these could be supplemented with quantitative data on the frequency of these consultation modalities.

### Effects on Vulnerable Populations

Certain populations remain underrepresented in remote consultation research due to gaps in data collection and reporting. For example, the voices of patients with dementia were reportedly lost in remote communications, which primarily engaged their caregivers instead [47]. In studies with recently arrived migrants [48,49], patients with language barriers or living in limited-income settings found remote consultations hard to engage with [50].

Moreover, one study reported how the introduction of home-schooling increased the skills of using videoconferencing technology among affluent families from urban and rural settings, which facilitated access to remote health care [43]; however, poorer families often had to share computer facilities and operate with less stable broadband networks. Nevertheless, a study in Northwest London showed that appointment attendance rates were similar between sociodemographic groups, regardless of whether remote or in-person, indicating that remote consultations have neither widened nor reduced inequality [28]. However, these results might be biased, as only patients who had the ability and need to receive remote consultations were offered this modality. Without comprehensive data on these vulnerable populations, health care services risk implementing policies that fail to address their unique needs.

### Gaps in Data Affecting Long-Term Evaluations

The lack of complete and standardized data hinders long-term evaluations of remote consultations. A key limitation of studies that investigated health care contacts by modality is that changes have primarily been studied early in the COVID-19 pandemic (up until September 2020), a time when many factors were affecting health care systems and uptake. Two studies explored changes in consultation rates over time in secondary mental health services [7] and primary care [20] between March-September and April-July 2020, respectively, with both reporting a decrease in face-to-face consultations.

Another study explored consultation rates and modalities in relation to socioeconomic deprivation from 2018 to 2022 [51]. The study used data from 397 GP practices in England and found that remote consultations increased from 0.91 on average per person-year in the period 2018‐2019 to 2.45 in 2020‐2021 and slightly decreased to 2.34 in 2021‐2022, suggesting changes in patient and clinician preferences. The study also showed that although remote consultations increased for all deprivation quintiles, it was larger for people in the most deprived quintile. However, inconsistencies in the recording consultation modality limited the ability to assess the reasons behind these changes, which could lead to an underestimation of the number of remote consultations. Similarly, a study analyzing NHS Digital data found that telephone consultations increased 3-fold between February 2020 and August 2021 [52]. People from low-income backgrounds were less likely to use remote consultations and receive same-day consultations at the beginning of the first COVID-19 lockdown in March 2020; however, these inequalities disappeared later in the pandemic period [52].

## Public Perceptions of Remote Consultations

While data sources allow an understanding of the uptake of remote consultations, the public’s perceptions could help put these findings in context. A content analysis of UK-based posts on the social media platform Twitter (subsequently rebranded X) [42] provided insights into patients’ opinions regarding remote consultations during the different stages of the COVID-19 pandemic. The proportion of comments with a positive tone was almost double during the initial stages of the pandemic (March-May 2020) compared to both the period before the pandemic (January 2018-February 2020) and when some social distancing restrictions were lifted later in the pandemic (June-October 2020). Twitter was a useful data source to understand how public attitudes toward remote consultations evolve as a result of the different stages in the pandemic.

Patients reported that the choice of consultation modality (face-to-face, video, or telephone) is valued and varies according to the reason for the consultation (eg, a preference for obtaining repeat prescriptions over the phone) [43,53,54]. Patients and clinicians reported their discomfort with a lack of patient choice during the pandemic [46,53,55] and a concern that remote consultations might be used as a default option for cost- and time-saving reasons [20,49,50,55]. While the restrictions did not allow flexibility at the time, clinicians and patients can assess the advantages and disadvantages of each consultation modality in order to tailor each consultation for different patient needs.

Issues in detecting and evaluating variations in communication quality were present, including uncertainties of whether and how patients’ queries were received and a lack of confidence using remote means of interaction [39,44]. Some patients reported that appropriate quality of care and relationships could only be built in person, in part due to the added value of nonverbal communication in face-to-face consultations [43-45]. Particular concerns were that remote consultations would increase responsibility on the part of patients to articulate their needs verbally (especially over telephone where patients cannot be seen) and reduce the availability of other informal or nonverbal avenues of communication provided in clinics and waiting rooms [39,42,44,45,49].

## Recommendations and Conclusions

This paper, focusing on England, highlights the urgent need to improve data quality to better understand the variations in implementation, uptake, and impact of the wide-scale introduction of remote consultations as they become embedded. Many of the data challenges presented have relevance beyond the English context (eg, lack of reliability and standardization in EHRs and diversity in populations and services) [56-58]. Addressing issues related to data standardization, completeness, and linkage will enable more reliable evaluations and support evidence-based decision-making.

Missing, inconsistent, and inaccurate data in EHRs could be addressed by simplifying and automatizing platforms or software to avoid adding an extra burden to clinicians when recording data. In addition, data sharing and standardization of variables and service definitions across platforms are a start to improving analytical processes and the reliability of outputs. Health care systems implementing remote consultations may find suggestions for improved data quality valuable while building or enhancing digital health care systems to ensure valid and reliable results from evaluations based on observational data. Furthermore, while linkage across platforms is a complex process, having standardized definitions across trusts could facilitate its outputs, which would positively impact the quality and representativeness of the data.

Qualitative evidence can help us understand barriers and facilitators to both patient and clinician affecting the use and uptake of remote consultations and may highlight data quality challenges (eg, poor quality of documentation and lack of information regarding choice). Due to safety reasons, remote consultations were the default option during the COVID-19 pandemic, but in a world that has become more familiar with remote interactions, understanding the patients’ experience with this modality is essential to ensure a patient-centered approach. Comparative mixed methods studies of individuals and groups (including both patients and staff) experiencing different levels of social advantage according to a variety of demographic, socioeconomic, and geographical (eg, urban vs rural) parameters are also recommended to measure, quantify, and unpick the mechanisms by which remote consultation engagement and uptake changes over time across England.

From the later stage of the pandemic, blended or hybrid approaches incorporating remote options have emerged, so evaluations need to consider whether and when alternatives to remote contact are available in a patient’s pathway. Decisions should also consider patient and service characteristics. Adding to complexity, but important for interpretation of outcomes, is service-level context on whether patients enter remote or nonremote pathways by their own choice or by that of clinicians or service planners, along with the factors used to guide such choices. Long-term and cost-effectiveness evaluations as well as the assessment of expected and unexpected benefits and adverse effects are needed to explore real-world impact and develop guidance for the future positioning of remote consultations. 

A glossary of key terms is provided in Textbox 1.

Textbox 1. **Glossary of key terms used in this article.**

- Consultations: Appointments between a health professional and a patient to diagnose or treat a health condition.
- Data sources: The origin of the information gathered for research, including databases, studies, and health records.
- Digital transformation: The process of integrating digital technology into all areas of a health care system, changing how services are delivered, and how care is provided.
- Electronic health record: A digital version of a patient’s paper chart, containing their medical history, treatment plans, and other health information.
- National Health Service: The publicly funded health care system in England that provides medical services to residents.
- Remote consultations: Medical appointments conducted via video, telephone, or digital platforms rather than in person.

## Abbreviations

EHR: electronic health record
GP: general practitioner
NHS: National Health Service
NLP: natural language processing
